# Comparative efficacy of different types of exercise modalities on psychiatric symptomatology in patients with schizophrenia: a systematic review with network meta-analysis

**DOI:** 10.1038/s41598-024-57081-3

**Published:** 2024-03-25

**Authors:** Wenlai Cui, Zhitao Liu, Cheng Liang, Zhizhi Zhao

**Affiliations:** 1 Graduate School, Capital University of Physical Education and Sports, Beijing, China; 2 School of Physical Education and Sport Science, Fujian Normal University, Fuzhou, Fujian China; 3 Institute of Physical Education and Training, Capital University of Physical Education and Sports, Beijing, China

**Keywords:** Neuroscience, Psychology, Diseases, Neurology

## Abstract

This network meta-analysis investigated the effects of 8 types of physical exercises on treating positive symptoms, negative symptoms, general psychopathology, and the Positive And Negative Syndrome Scale (PANSS) total score in patients with schizophrenia. The methods adhered to PRISMA guidelines and used the Cochrane risk of bias tool for quality assessment, and Stata software for data analysis. Data were sourced from PubMed, Embase, Web of Science, and the Cochrane database up to August 15, 2023, following PICOS principles. A total of 25 studies including 1441 participants were analyzed. Results showed that resistance exercise seems to be effective for improving positive symptoms, while Yoga was more effective for negative symptoms. Low-intensity aerobic exercise was optimal for general psychopathology, and Yoga was effective in improving the PANSS total score. The study concluded that yoga and aerobic exercise demonstrated superior performance, but the impact of exercise on patients with schizophrenia is also influenced by individual factors and intervention dosages. Therefore, a pre-assessment of patients considering factors such as interests, hobbies, and physical capabilities is crucial for selecting appropriate exercise modalities.

## Introduction

According to the survey results from the World Health Organization (WHO) in 2022, approximately 24 million people worldwide are affected by schizophrenia. This condition predominantly impacts individuals between the ages of 20 and 35, with males often experiencing onset at an earlier age than females^1,2^. Concerningly, about 50% of patients will endure some degree of symptoms throughout their lifetime^3,4^. Schizophrenia not only profoundly influences the quality of life for affected individuals but also manifests in a shorter average life expectancy and a higher suicide rate^5,6^. As of 2015, over 17,000 individuals had lost their lives due to schizophrenia or related disorders and behaviors^7^. Faced with this reality, the prevention and treatment of schizophrenia emerge as urgent and crucial issues requiring our attention.

The treatment of schizophrenia primarily emphasizes the comprehensive use of antipsychotic medications, psychotherapy, and social support, among other approaches^8^. However, research indicates suboptimal performance of drug therapy in addressing negative symptoms^9^. Additionally, frequent use of antipsychotic medications may lead to a range of harmful side effects^10^, such as extrapyramidal symptoms, weight gain, and delayed movement disorders. Consequently, the significance of non-pharmacological treatments in schizophrenia management is gaining prominence, particularly with interventions like exercise^11,12^.

Exercise interventions have become a crucial and cost-effective approach in the treatment of schizophrenia due to their low cost and ease of implementation^13,14^. Numerous randomized controlled trials have investigated the impact of various types of exercise interventions on individuals with schizophrenia, including yoga, tai chi, dance, aerobic exercise, cycling, and more^15–18^. These studies have explored multiple aspects, including cognitive function, quality of life, and physical fitness^19–21^. Further literature reviews emphasize the positive effects of physical activity on the treatment of schizophrenia^11^. Specifically, research suggests that yoga, as a mind–body practice, may offer various potential benefits for individuals with schizophrenia^22^, and there is support for the positive effects of aerobic exercise on addressing cognitive deficits in schizophrenia treatment^23^. Meta-analysis studies further confirm that physical activity helps alleviate depressive symptoms and increases the level of physical activity in patients^24,25^. Moreover, some studies indicate that aerobic exercise and mind–body exercises significantly impact the negative symptoms of schizophrenia^26,27^. Resistance exercise also played a positive role in improving symptoms of schizophrenia^28^. Overall, these findings underscore the effectiveness of exercise interventions as a treatment for schizophrenia.

There is a diverse range of exercise intervention modalities, including yoga, dance, tai chi, Baduanjin, aerobic exercise, and more. However, there is still uncertainty about which exercise intervention is most beneficial for schizophrenia. Therefore, this study conducted a network meta-analysis (NMA) of randomized controlled trials on exercise interventions for patients with schizophrenia. The aim is to provide valuable information for selecting the optimal exercise modality in the treatment of schizophrenia.

## Materials and methods

### Protocol and registration

This systematic review and meta-analysis adhere to the guidelines established by the Cochrane Handbook and are reported in accordance with the Preferred Reporting Items for Systematic Reviews and Meta-Analyses (PRISMA) statement^29,30^. The NMA has been registered with PROSPERO (CRD42023452874).

### Search strategy

In accordance with the PICOS framework, we conducted systematic searches in the PubMed, Embase, Web of Science, and Cochrane databases, covering the period from database inception through August 15, 2023. The search strategy focused primarily on aspects related to the study population, intervention methods, and research methodology. The search terms and keywords used are as follows: (“Exercise” or “Exercises” or “Sports” or “Training” or “Physical Activity” or “Acute Exercise” or “Aerobic exercise” or “Isometric Exercises” or “Nerve Exercise” or “Leisure Activities” or “Endurance” or “Resistance” or “Flexibility” or “Sports games” or “Running” or “Bicycles” or “Gymnastics” or “Jump rope” or “Dance” or “Tai Chi” or “Yoga” or “Ball Sports” or “Racquet Sports” or “Water Sports” or “Swimming”) AND (“Schizophrenia” or “Schizophrenias” or “Schizophrenic Disorders” or “Disorder, Schizophrenic” or “Disorders, Schizophrenic” or “Schizophrenic Disorder” or “Dementia Praecox”) AND (“Randomized controlled trial” or “controlled clinical trial” or “randomized” or “placebo” or “randomly”). For the detailed search strategy, please refer to Appendix S1. Additionally, we manually reviewed the bibliographies of relevant reviews and supplemented our research with identified articles. If necessary, we reached out to study authors for further information.

### Inclusion and exclusion criteria

To ensure the scientific rigor and comparability of the study, clear inclusion and exclusion criteria were employed. All included studies had to meet the following conditions:

- Published randomized controlled trials to ensure high-quality research design and methods;
- Participants must be individuals diagnosed with schizophrenia to maintain the focus and applicability of the study;
- Experimental interventions must include a component of exercise to ensure the relevance of the study's interventions to physical activity;
- The intervention in the control group should be non-pharmacological (such as standard treatment, alternative intervention, placebo, usual care, stretching, etc.), and should be able to provide valid outcome data.
- Outcome measures must include scores from the Positive And Negative Syndrome Scale (PANSS) or include the Scale for Assessment of Positive Symptoms (SAPS) or Scale for Assessment of Negative Symptoms (SANS) to maintain a comprehensive assessment of schizophrenia symptoms.

Exclusion criteria encompassed:

- Non-original research literature such as study reports, conference abstracts, reviews, etc., to ensure that included studies provided sufficient detailed data;
- Observational and cross-sectional studies to ensure that included studies had an experimental nature;
- Duplicate experimental data from multiple publications of the same study to avoid introducing redundant information in the analysis.

### Data extraction

Two authors (WLC and ZTL) independently screened the titles and abstracts of literature to identify those meeting the inclusion criteria. If either author considered a study eligible based on the criteria, the full text of the article was obtained. Subsequently, the full texts were independently assessed by the two authors to determine compliance with the requirements. In cases of disagreement, a group discussion was conducted, and consensus was reached through the discussion. This study did not impose restrictions on participant age, gender, body mass index, publication date, or language.

Two authors (CL and ZZZ) independently extracted data from the included studies. We designed an Excel spreadsheet to capture relevant data, including publication characteristics (title, author names, publication year, country), methodological features (number of study groups, design of each group, interventions, sample size), participant characteristics (age, gender ratio, duration of illness), risk assessment features, and outcome characteristics.

During the extraction of outcome data, if post-intervention result data were presented graphically without explicit textual explanation, we utilized Engauge Digitizer software for data extraction. For studies with multiple follow-ups, we only extracted data from the immediate post-intervention assessments.

### Quality assessment

Two pairs of authors (WLC and ZTL, CL and ZZZ) conducted quality assessment of the included literature using the quality assessment criteria recommended by the Cochrane systematic review^31^. All studies included in this review were randomized controlled trials. As per the Cochrane Handbook, for inclusion of randomized controlled trials, the recommended tool is the revised version of the Cochrane tool, known as the Risk of Bias tool (Rob 2)^32^. The Rob 2 tool offers a framework for assessing the risk of bias in individual outcomes within various types of randomized trials. The assessment criteria comprise seven domains: random sequence generation, allocation concealment, blinding of participants and personnel, blinding of outcome assessment, incomplete outcome data, selective reporting, and other biases. According to the Cochrane Handbook, reviewers assign a risk of bias rating for each domain in different studies, with bias risk categorized into three levels: “low risk,” “some concerns,” and “high risk.” If all domains are assessed as having a “low risk” of bias, the overall bias risk is considered “low.” If some domains are assessed as having “some concerns” and no domains are rated as “high risk,” the overall bias risk is categorized as “some concerns.” However, if any single domain is assessed as having a “high risk” of bias, the overall bias risk is classified as “high”^33^.

### Statistical analysis

In the included randomized controlled studies involving individuals with schizophrenia, all variables were continuous and expressed as mean with standard deviations (SD). For other forms of data presentation, conversion procedures were applied. Continuous variables in this study were reported as mean differences (MD), with a 95% confidence interval (95% CI) for analysis. Recognizing inherent differences among studies, a random-effects model was employed for analysis.

Following the PRISMA NMA guidelines, a Bayesian framework was utilized in conjunction with Stata 16.0 software for Markov Chain Monte Carlo simulation aggregation and analysis of NMA data^34,35^. The node-splitting method was adopted to quantify and verify consistency between direct and indirect comparisons. Subsequent to computation using Stata software commands, a consistency test was performed^36^, whereby a P-value greater than 0.05 indicated successful consistency.

A Bayesian model was employed for NMA. The data were preprocessed using the “network group” command, and a grid of evidence plot was generated. In the network evidence plot, each intervention type was represented by a point, and the size of the point was proportional to the sample size of participants involved in the intervention studies. Connecting lines between two points denoted direct comparisons between the two intervention modalities, and the width of the line was proportional to the number of included studies^37^. The Surface Under the Cumulative Ranking (SUCRA) curve was used to rank the effects of different exercise modalities, and the cumulative ranking probabilities were depicted in a table. Additionally, to assess the impact of publication bias and small-study effects, a network funnel plot was constructed, and visual inspection was conducted to evaluate symmetry. Given the potential for bias, Egger’s and Begg’s tests were performed; a P-value greater than 0.05 indicated the absence of significant publication bias.

### Classification of intervention methods

The intervention measures included in the study are Yoga, power cycling, treadmill exercise, Tai chi, stretching, resistance exercise, etc. Based on the included intervention measures, we classified these intervention measures into 8 categories: Yoga, medium and high-intensity aerobic exercise (MHAE), low-intensity aerobic exercise (LAE), resistance exercise (RE), stretching exercise (SE), Chinese traditional sports (CTS), Multi-mode movement (MM), and treatment-as-usual (TAU).

For the Yoga intervention type, we did not make a detailed division, it could be gentle yoga, power yoga, or chair yoga, among others. RE refers to an exercise method that strengthens muscle strength, endurance, and muscle mass through the use of external resistance (such as weightlifting, elastic bands, or body weight). MHAE and LAE are divided based on the type of exercise and exercise intensity. First, based on exercise intensity, if the intervention measure reaches 60% or more of the maximum heart rate, aerobic capacity, or peak heart rate of the subjects, we consider it as MHAE, below 60% is considered LAE. Second, when exercise intensity is not explicitly stated, we classify activities such as power cycling and treadmill exercises as MHAE, and activities such as walking and jogging as LAE. CTS includes a series of physical sports and fitness activities derived from traditional Chinese culture and lifestyle. In this study, CTS includes Tai chi, Qigong, martial arts, Chinese Kung Fu, Tai chi sword, Eight-section Brocade, etc. Other types of exercises include stretching exercises and multi-mode movement. MM refers to a comprehensive exercise mode that combines multiple different exercise forms or training methods. In this paper, if the exercise type is not explicitly stated or includes multiple exercise modalities in the intervention measure, we classified it as MM.

## Results

### Study selection

**Figure 1:**
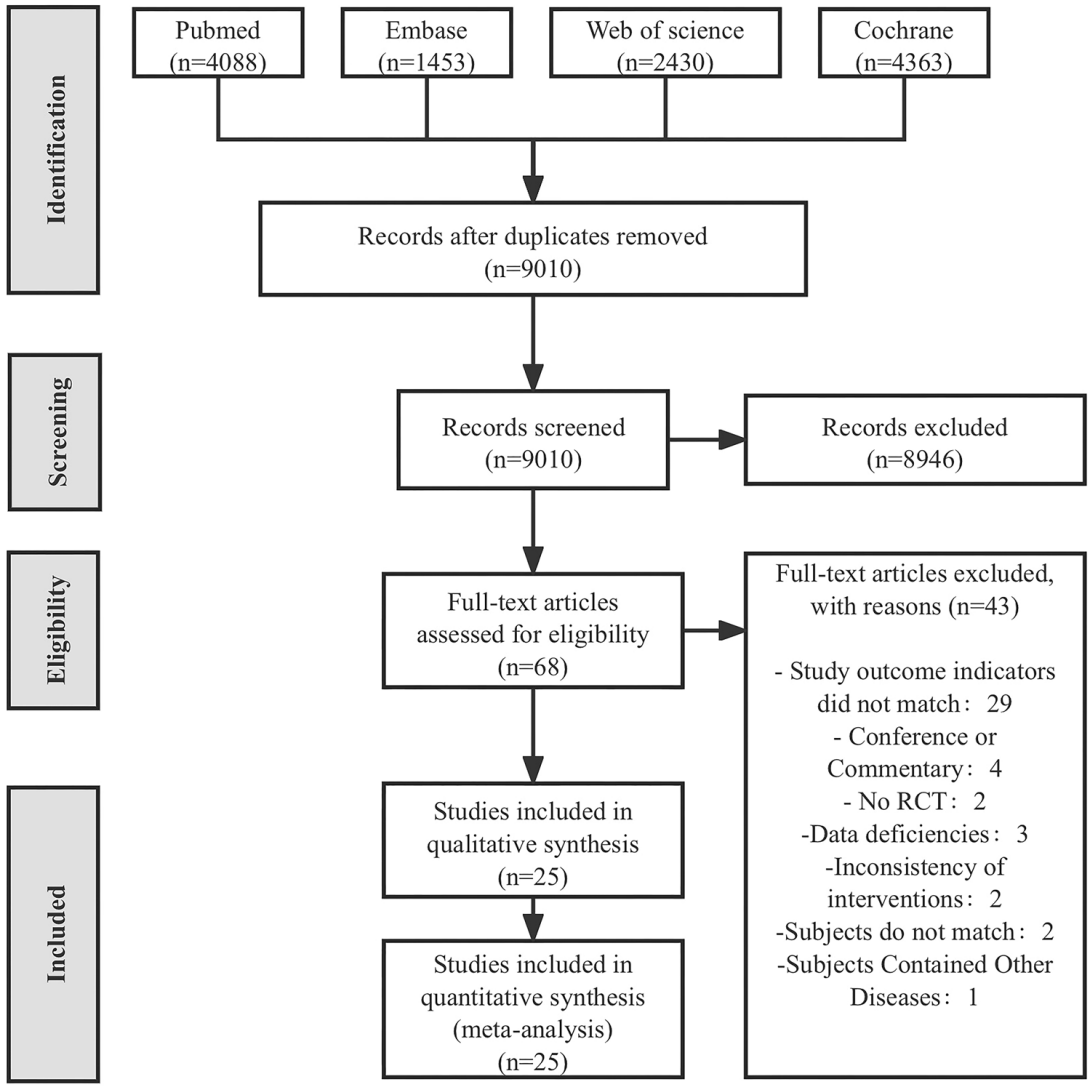
PRISMA study flow diagram.

A total of 12,334 articles were retrieved from four databases (PubMed: 4088, Embase: 1453, Web of Science: 2430, Cochrane: 4363). After removing duplicate articles, 9010 remained. Following the review of titles and abstracts, 68 articles were retained. Upon thorough examination of the full texts, 25 articles were ultimately included (Fig. 1).

### Study characteristics

After screening, a total of 25 articles were finally included^28,38–61^, and Table 1 displays the characteristics of the included studies. The publication years of the included studies ranged from 2007 to 2023. In terms of the distribution of countries among the included studies, India had the highest number with 6 articles, followed by Japan with 5 studies. Regarding the included study groups, the 25 articles comprised a total of 52 groups, with 2 articles including 3 groups.

**Table 1:** Basic characteristics of the study.

Study	Country	N		M/F		Age (years)		Duration of illness		Length of intervention
		EG	CG	EG	CG	EG	CG	EG	CG	
T. Mullapudi, 2023	India	21	20	14/7	13/7	25.10 ± 7.37	27.55 ± 7.93	7.29 ± 4.71(y)	8.53 ± 5.08(y)	6 Months
N. M. Khonsari, 2022	Iran	20	20	12/8	9/11	32.7 ± 8.6	37.2 ± 7.8	10.3 ± 4.4(y)	11.5 ± 4.5(y)	8 Weeks
Y. Kurebayashi, 2022	Japan	4	14	NR/NR	NR/NR	50.3 ± 14.0	59.7 ± 13.0	31.0 ± 10.2(y)	30.9 ± 15.1(y)	8 Weeks
N. P. Rao, 2021	India	45	44	29/16	32/12	34.79 ± 7.06	33.65 ± 8.95	137.42 ± 83.59(m)	125.82 ± 90.71(m)	12 Weeks
N. Massa, 2020	USA	9	6	9/0	6/0	53.44 ± 7.14	52.67 ± 10.07	NR	NR	12 Weeks
M. Li, 2020	China	30	31	24/6	23/8	51.00 ± 6.86	50.97 ± 8.54	21.63 ± 10.13(y)	19.84 ± 10.47(y)	24 Weeks
T. Shimada, 2019	Japan	16	15	NR/NR	NR/NR	NR	NR	NR	NR	12 Weeks
A. J. Romain, 2019	Canada	27	25	25/13	16/12	29.70 ± 7.24	32.12 ± 7.10	NR	NR	6 Months
P. W. Wang, 2018	China	25	23	15/18	15/14	38.3 ± 8.34	38.72 ± 8.62	NR	NR	3 Months
D. Curcic, 2017	Serbia	40	40	23/17	19/21	39.95 ± 9.51	41.75 ± 9.45	12.52 ± 7.62(y)	11.14 ± 7.21(y)	12 Weeks
S. Ikai, 2017	Japan	28	28	18/10	18/10	55.5 ± 11.4	55.0 ± 15.8	23.4 ± 14.4(y)	28.9 ± 14.8(y)	12 Weeks
C. Y. Su, 2016	China	22	22	10/12	10/12	37.64 ± 8.23	36.68 ± 8.33	14.05 ± 8.77 (y)	12.00 ± 7.65(y)	3 Months
R. Kang, 2016	China	118	126	53/65	63/63	46.4 ± 11.9	45.4 ± 12.3	21.3 ± 11.7(y)	19.8 ± 12.1(y)	12 Months
A. Kaltsatou, 2015	USA	16	15	14/2	11/4	59.5 ± 19.6	60.4 ± 8.6	35.1 ± 19.1(y)	33.7 ± 19.3(y)	8 Months
S. Y. Loh, 2016	Malaysia	52	48	35/17	39/13	46.0 ± 14	53.00 ± 11	15.5 ± 12(y)	25.00 ± 12(y)	3 Months
S. Ikai, 2014	Japan	25	25	16/9	17/8	53.5 ± 9.9	48.2 ± 12.3	25.3 ± 9.6(y)	24.7 ± 11.1(y)	8 Weeks
S. Ikai, 2013	Japan	25	24	16/9	16/8	54.8 ± 9.0	51.5 ± 15.1	24.5 ± 10.8(y)	27.7 ± 11.5(y)	8 Weeks
T. W. Scheewe, 2013	Netherlands	20	19	23/8	23/9	29.2 ± 7.2	30.1 ± 7.7	2302.5 ± 2056.5(d)	2540.1 ± 2233.2(d)	6 Months
S. Varambally, 2013	India	35	25	26/18	23/21	31.7 ± 8.8	31.1 ± 7.8	119.5 ± 102(m)	97.3 ± 90.8(m)	6 Weeks
S. Varambally, 2012	India	46	37	28/18	27/10	32.8 ± 10.0	33.6 ± 9.5	129.7 ± 96.6(m)	121.5 ± 105.8(m)	4 Months
		36		28/8		30.6 ± 7.3		88.6 ± 91.9(m)		
J. Heggelund, 2011	Norway	12	7	9/3	4/3	30.5 ± 8.7	38.9 ± 11.4	35.2 ± 19.6(m)	70.1 ± 62.9(m)	8 Weeks
E. Visceglia, 2011	USA	9	9	12/6		37.40 ± 13.73	48.13 ± 11.24	31.40 ± 20.38(m)	53.00 ± 76.80(m)	8 Weeks
R. V. Behere, 2011	India	27	22	18/9	15/7	31.3 ± 9.3	33.6 ± 9.9	126.2 ± 101.6(m)	121.6 ± 108.6(m)	4 Months
		17		14/3		30.2 ± 8.0		86.6 ± 93.1(m)		
A. A. Acil, 2008	Turkey	15	15	NR/NR	NR/NR	32.06	32.66	10.93(y)	9.60(y)	10 Weeks
G. Duraiswamy, 2007	India	21	20	19/12	23/7	32.53 ± 7.9	31.30 ± 7.9	99.1 ± 96.1(m)	81.1 ± 81.4(m)	4 Months

*M/F* male/female, *EG* experimental group, *CG* control group, *NR* no report, *Y* years, *M* months, *D* days.

Regarding the baseline information of the study participants, the baseline of the included studies included 1564 participants. In the final sample, there were a total of 1441 participants, with 761 participants in the experimental groups and 680 participants in the control groups. The sample size in the experimental groups ranged from 4 to 118, while in the control groups, it ranged from 6 to 126. One article did not report the age range of the participants; excluding this article, the age range in the experimental groups was approximately 18 to 80 years, and in the control groups, it was approximately 19 to 69 years. Two articles did not report the gender ratio of the participants; excluding these two articles, the baseline sample included 912 male participants and 554 female participants. Four articles did not report the duration of illness for the participants; excluding these four articles, the duration of illness for participants in the experimental groups ranged from 1 to 55 years, and for participants in the control groups, it ranged from 0 to 53 years.

In terms of the outcome measures included in the studies, among the 24 articles, the outcome measures included Positive Symptoms and Negative Symptoms; for 15 articles, the outcome measures included General Psychopathology; and for 17 articles, the outcome measures included PANSS total (Table 2).

**Table 2:** Study intervention and outcome reporting characteristics.

Study	Interventions		Type		Frequency (times/week)	Duration	PS	NS	GP	PT
	EG	CG	EG	CG						
T. Mullapudi, 2023	Yoga	TUA	Yoga	TUA	≥ 3	NR	✓	✓		
N. M. Khonsari, 2022	Power bike/invisible jump rope	TUA	MHAE	TUA	3	40 min	✓	✓		✓
Y. Kurebayashi, 2022	Aerobics, form of your choice (60% HRmax)	TUA	MHAE	TUA	2	60 min	✓	✓	✓	✓
N. P. Rao, 2021	Yoga	TUA	Yoga	TUA	3	60 min	✓	✓		
N. Massa, 2020	Power bike (50–80% HRmax)	Stretching and Balancing Exercises	MHAE	SE	3	60 min	✓	✓		✓
M. Li, 2020	Baduanjin	Brisk walking	CTS	LAE	5	40 min				✓
T. Shimada, 2019	Treadmills/power bike (60–80% aerobic capacity)	TUA	MHAE	TUA	2	60 min	✓	✓	✓	✓
A. J. Romain, 2019	Treadmill interval training (80% HRmax)	TUA	MHAE	TUA	2	30 min	✓	✓	✓	
P. W. Wang, 2018	AE	Flexibility, stretching exercises	MHAE	SE	5	40 min	✓	✓	✓	✓
D. Curcic, 2017	Brisk walking or jogging (60–70% HRmax)	TUA	MHAE	TUA	4	45 min	✓	✓	✓	✓
S. Ikai, 2017	Yoga	TUA	Yoga	TUA	2	20 min	✓	✓	✓	✓
C.Y. Su, 2016	Treadmill exercise (55–69% HRmax)	Flexibility, stretching exercises	MHAE	SE	3	40 min	✓	✓		
R. Kang, 2016	Tai Chi	TUA	CTS	TUA	0.5	45 min	✓	✓	✓	✓
A. Kaltsatou, 2015	Traditional dances	TUA	CTS	TUA	NR	NR	✓	✓	✓	✓
S. Y. Loh, 2016	Walking	TUA	LAE	TUA	3	20–40 min	✓	✓	✓	
S. Ikai, 2014	Yoga	TUA	Yoga	TUA	1	60 min	✓	✓	✓	✓
S. Ikai, 2013	Yoga	TUA	Yoga	TUA	1	60 min	✓	✓	✓	✓
T. W. Scheewe, 2013	Muscle strength exercises	Cognitive activities	RE	TUA	2	60 min	✓	✓		✓
S. Varambally, 2013	Yoga	Sporting activity	Yoga	MM	5	60 min	✓	✓	✓	✓
S. Varambally, 2012	Yoga	TUA	Yoga	TUA	6–7	45 min	✓	✓	✓	✓
	Exercise		MM		6–7	45 min				
J. Heggelund, 2011	Treadmill exercise (85–95% HRmax)	Cognitive activities	MHAE	TUA	3	25 min	✓	✓	✓	✓
E. Visceglia, 2011	Yoga	TUA	Yoga	TUA	2	45 min	✓	✓	✓	✓
R. V. Behere, 2011	Yoga	TUA	Yoga	TUA	NR	60 min	✓	✓		
	Sporting activity		MM		NR	60 min				
A. A. Acil, 2008	Aerobics	TUA	LAE	TUA	3	40 min	✓	✓		
G. Duraiswamy, 2007	Yoga	Brisk walking, jogging, standing and sitting and relaxation exercises	Yoga	LAE	5	60 min	✓	✓		✓

EG, experimental group; CG, control group; PS, Positive Symptoms; NP, Negative Symptoms; GP, General Psychopathology; PT, PANSS total; MHAE, medium and high-intensity aerobic exercise, LAE, low-intensity aerobic exercise, RE, resistance exercise, SE, stretching exercise, CTS, Chinese traditional sports, MM, Multi-mode movement, TAU, treatment-as-usual.

### Risk of bias

**Figure 2:**
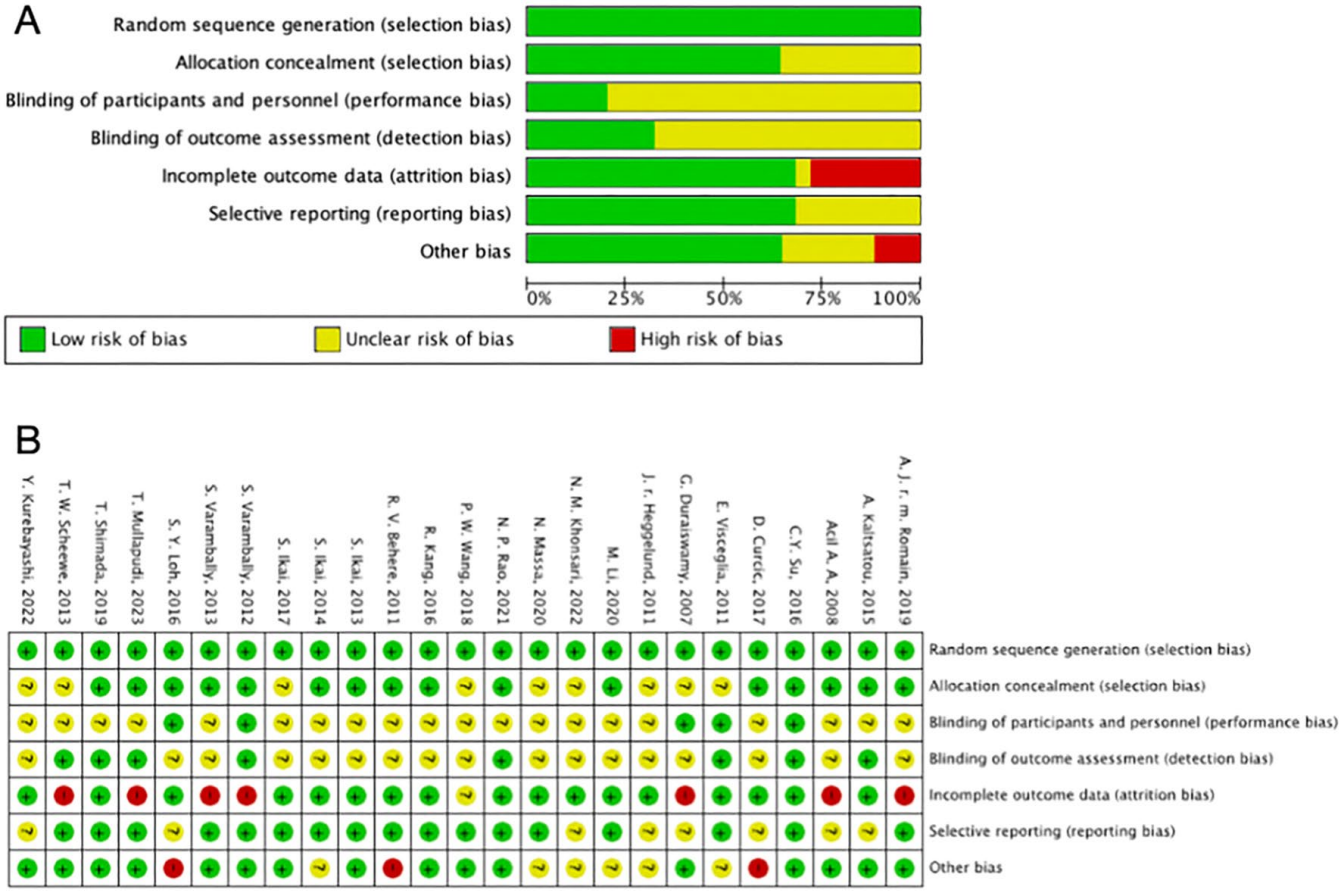
Risk of bias summary: (**A**) Methodological quality of included studies. (**B**) The distribution of the methodological quality of included studies.

All studies considered the risk of low bias in generating random sequences. Among the 25 articles, 16 were deemed to have a low risk of allocation concealment bias, while 9 did not specify their allocation concealment methods and were considered to have an uncertain risk. There was a higher risk of bias in blinding for both researchers and participants because implementing exercise interventions under double-blind conditions is challenging, resulting in an overall higher risk of bias for this indicator. In terms of outcome assessment blinding, 8 studies employed professional physicians or blinded assessors for outcome assessment, indicating a low risk, while 17 studies did not mention the outcome assessment method, posing a certain risk. Seventeen studies had consistent or essentially consistent post-intervention participant numbers with baseline, and they had complete outcome reporting, considered to have a low risk. Eight studies had some discrepancies in post-intervention numbers compared to baseline, indicating a certain risk. The risk of selective reporting bias was deemed low in 17 studies, while 8 studies were considered to have some risk because they did not report pre-registered protocols or did not provide detailed reasons for participant dropout. Four studies had some risk of other biases (Fig. 2).

### NMA

**Figure 3:**
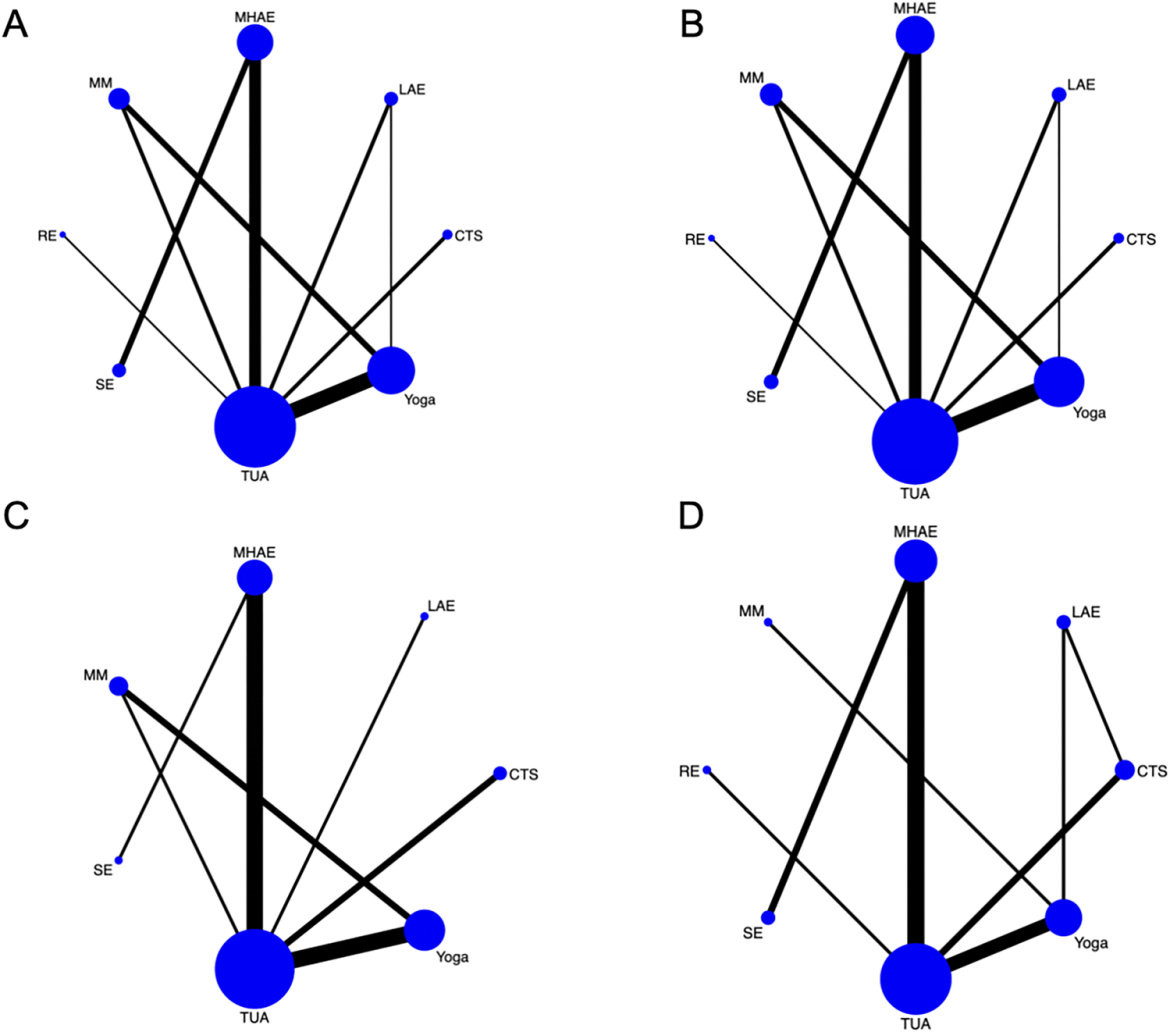
(**A**) NMA figure for positive symptoms. (**B**) NMA figure for negative symptoms. (**C**) NMA figure for general psychopathology. (**D**) NMA figure for PANSS total.

The complete NMA diagram, as shown in Fig. 3, represents interventions for the treatment of schizophrenia. Nodes represent different intervention measures, and we conducted comparisons among them. When there are differences in outcomes, variations may exist in the comparisons between intervention measures. The width of the connecting lines indicates the number of studies comparing the interventions, with wider lines representing a greater number of comparative studies between the interventions.

#### Positive symptoms

A total of 24 studies reported on Positive Symptoms, involving 8 different intervention measures. Initially, we assessed the consistency and inconsistency of direct and indirect comparisons among the 24 studies. The results indicated that almost all P-values were greater than 0.05, with a total P-value of 0.4552, suggesting that the consistency among the studies is acceptable.

**Figure 4:**
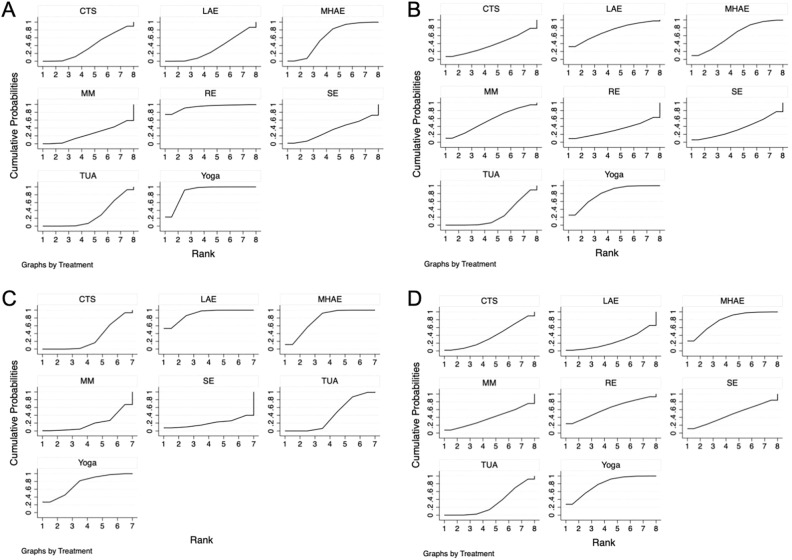
(**A**) SUCRA plot for positive symptoms. (**B**) SUCRA plot for negative symptoms. (**C**) SUCRA plot for general psychopathology. (**D**) SUCRA plot for PANSS total.

The statistical significance results of the NMA are as follows (Table 3): Yoga [MD: − 2.12, 95% CI: (− 3.08, − 1.16)] and RE [MD: − 3.20, 95% CI: (− 6.21, − 0.18)] showed better improvement in Positive Symptoms compared to TUA. Additionally, the therapeutic effect of Yoga was superior to CTS [MD: − 1.91, 95% CI: (− 3.52, − 0.29)], LAE [MD: − 2.06, 95% CI: (− 3.45, − 0.66)], and MM [MD: − 2.37, 95% CI: (− 4.07, − 0.67)]. In terms of SUCRA, RE ranked first in the probability of affecting Positive Symptoms (SUCRA: 93.4%) (Fig. 4A).

**Table 3:** League table on Positive Symptoms.

RE	Yoga	MHAE	CTS	LAE	SE	TUA	MM
RE	1.08 (− 2.08, 4.25)	2.38 (− 0.79, 5.55)	2.99 (− 0.22, 6.20)	3.12 (− 0.60, 6.84)	3.14 (− 0.04, 6.31)	3.20 (0.18, 6.21)	3.45 (− 0.09, 7.00)
− 1.08 (− 4.25, 2.08)	Yoga	1.30 (− 0.24, 2.84)	1.91 (0.29, 3.52)	2.04 (− 0.44, 4.51)	2.06 (0.66, 3.45)	2.12 (1.16, 3.08)	2.37 (0.67, 4.07)
− 2.38 (− 5.55, 0.79)	− 1.30 (− 2.84, 0.24)	MHAE	0.61 (− 0.67, 1.90)	0.74 (− 1.21, 2.69)	0.76 (− 0.59, 2.11)	0.82 (− 0.16, 1.81)	1.08 (− 1.13, 3.29)
− 2.99 (− 6.20, 0.22)	− 1.91 (− 3.52, − 0.29)	− 0.61 (− 1.90, 0.67)	CTS	0.13 (− 2.21, 2.47)	0.15 (− 1.30, 1.60)	0.21 (− 0.90, 1.32)	0.46 (− 1.80, 2.73)
− 3.12 (− 6.84, 0.60)	− 2.04 (− 4.51, 0.44)	− 0.74 (− 2.69, 1.21)	− 0.13 (− 2.47, 2.21)	LAE	0.02 (− 2.35, 2.39)	0.08 (− 2.10, 2.26)	0.34 (− 2.60, 3.28)
− 3.14 (− 6.31, 0.04)	− 2.06 (− 3.45, − 0.66)	− 0.76 (− 2.11, 0.59)	− 0.15 (− 1.60, 1.30)	− 0.02 (− 2.39, 2.35)	SE	0.06 (− 0.94, 1.06)	0.32 (− 1.80, 2.44)
− 3.20 (− 6.21, − 0.18)	− 2.12 (− 3.08, − 1.16)	− 0.82 (− 1.81, 0.16)	− 0.21 (− 1.32, 0.90)	− 0.08 (− 2.26, 2.10)	− 0.06 (− 1.06, 0.94)	TUA	0.25 (− 1.61, 2.11)
− 3.45 (− 7.00, 0.09)	− 2.37 (− 4.07, − 0.67)	− 1.08 (− 3.29, 1.13)	− 0.46 (− 2.73, 1.80)	− 0.34 (− 3.28, 2.60)	− 0.32 (− 2.44, 1.80)	− 0.25 (− 2.11, 1.61)	MM

#### Negative symptoms

Similarly, 24 studies reported on Negative Symptoms, involving 8 different intervention measures. Initially, we assessed the consistency and inconsistency of direct and indirect comparisons among the 20 studies. The results indicated that almost all P-values were greater than 0.05, with a total P-value of 0.1993, suggesting that the consistency among the studies is acceptable.

The statistical significance results of the NMA are as follows (Table 4): Compared to TUA, Yoga [MD: = − 5.00, 95% CI: (− 8.66, − 1.35)] was more effective in improving Negative Symptoms. The SUCRA rankings for different intervention measures in improving Negative Symptoms were as follows: Yoga (79.4%) > LAE (72.0%) > MHAE (62.1%) (Fig. 4B).

**Table 4:** League table on negative symptoms.

Yoga	LAE	MHAE	MM	CTS	RE	TUA	SE
Yoga	0.33 (− 6.78, 7.43)	1.79 (− 4.05, 7.64)	2.17 (− 3.43, 7.77)	4.32 (− 4.25, 12.89)	4.57 (− 4.21, 13.35)	5.60 (− 5.65, 16.85)	5.00 (1.35, 8.66)
− 0.33 (− 7.43, 6.78)	LAE	1.47 (− 6.86, 9.79)	1.85 (− 6.88, 10.57)	3.99 (− 6.41, 14.40)	4.24 (− 6.34, 14.83)	5.28 (− 7.43, 17.98)	4.68 (− 2.26, 11.61)
− 1.79 (− 7.64, 4.05)	− 1.47 (− 9.79, 6.86)	MHAE	0.38 (− 7.08, 7.85)	2.53 (− 6.45, 11.51)	2.78 (− 3.78, 9.34)	3.81 (− 7.76, 15.38)	3.21 (− 1.33, 7.75)
− 2.17 (− 7.77, 3.43)	− 1.85 (− 10.57, 6.88)	− 0.38 (− 7.85, 7.08)	MM	2.15 (− 7.60, 11.90)	2.40 (− 7.54, 12.33)	3.43 (− 8.75, 15.61)	2.83 (− 3.09, 8.75)
− 4.32 (− 12.89, 4.25)	− 3.99 (− 14.40, 6.41)	− 2.53 (− 11.51, 6.45)	− 2.15 (− 11.90, 7.60)	CTS	0.25 (− 10.87, 11.37)	1.28 (− 11.88, 14.45)	0.68 (− 7.07, 8.43)
− 4.57 (− 13.35, 4.21)	− 4.24 (− 14.83, 6.34)	− 2.78 (− 9.34, 3.78)	− 2.40 (− 12.33, 7.54)	− 0.25 (− 11.37, 10.87)	RE	1.03 (− 12.27, 14.33)	0.43 (− 7.54, 8.41)
− 5.60 (− 16.85, 5.65)	− 5.28 (− 17.98, 7.43)	− 3.81 (− 15.38, 7.76)	− 3.43 (− 15.61, 8.75)	− 1.28 (− 14.45, 11.88)	− 1.03 (− 14.33, 12.27)	TUA	− 0.60 (− 11.24, 10.04)
− 5.00 (− 8.66, − 1.35)	− 4.68 (− 11.61, 2.26)	− 3.21 (− 7.75, 1.33)	− 2.83 (− 8.75, 3.09)	− 0.68 (− 8.43, 7.07)	− 0.43 (− 8.41, 7.54)	0.60 (− 10.04, 11.24)	SE

#### General psychopathology

In the case of General Psychopathology, 15 studies reported outcomes, involving 7 different intervention measures. Initially, we assessed the consistency and inconsistency of direct and indirect comparisons among the 15 studies. The results indicated that almost all P-values were greater than 0.05, with a total P-value of 0.7924, suggesting that the consistency among the studies is acceptable.

The statistical significance results of the NMA are as follows (Table 5): Compared to TUA, LAE [MD: − 2.50, 95% CI: (− 3.49, − 1.51)] and MHAE [MD: − 1.98, 95% CI: (− 2.71, − 1.24)] showed better therapeutic effects on General Psychopathology. Additionally, LAE [MD: − 2.70, 95% CI: (− 3.79, − 1.60)] and MHAE [MD: − 2.17, 95% CI: (− 3.04, − 1.30)] were more effective than CTS. The therapeutic effect of Yoga [MD: − 3.10, 95% CI: (− 5.56, − 0.63)] was superior to MM. In terms of SUCRA, LAE ranked first in the probability of affecting General Psychopathology (SUCRA: 89.5%) (Fig. 4C).

**Table 5:** League table on general psychopathology.

LAE	MHAE	Yoga	TUA	CTS	MM	SE
LAE	0.52 (− 0.71, 1.76)	0.74 (− 2.14, 3.62)	2.50 (1.51, 3.49)	2.70 (1.60, 3.79)	3.84 (0.11, 7.56)	5.06 (− 2.24, 12.37)
− 0.52 (− 1.76, 0.71)	MHAE	0.21 (− 2.59, 3.01)	1.98 (1.24, 2.71)	2.17 (1.30, 3.04)	3.31 (− 0.35, 6.98)	4.54 (− 2.66, 11.74)
− 0.74 (− 3.62, 2.14)	− 0.21 (− 3.01, 2.59)	Yoga	1.76 (− 0.94, 4.46)	1.96 (− 0.78, 4.70)	3.10 (0.63, 5.56)	4.33 (− 3.40, 12.05)
− 2.50 (− 3.49, − 1.51)	− 1.98 (− 2.71, − 1.24)	− 1.76 (− 4.46, 0.94)	TUA	0.20 (− 0.27, 0.66)	1.34 (− 2.25, 4.93)	2.56 (− 4.67, 9.80)
− 2.70 (− 3.79, − 1.60)	− 2.17 (− 3.04, − 1.30)	− 1.96 (− 4.70, 0.78)	− 0.20 (− 0.66, 0.27)	CTS	1.14 (− 2.48, 4.76)	2.37 (− 4.88, 9.62)
− 3.84 (− 7.56, − 0.11)	− 3.31 (− 6.98, 0.35)	− 3.10 (− 5.56, − 0.63)	− 1.34 (− 4.93, 2.25)	− 1.14 (− 4.76, 2.48)	MM	1.23 (− 6.85, 9.30)
− 5.06 (− 12.37, 2.24)	− 4.54 (− 11.74, 2.66)	− 4.33 (− 12.05, 3.40)	− 2.56 (− 9.80, 4.67)	− 2.37 (− 9.62, 4.88)	− 1.23 (− 9.30, 6.85)	SE

#### PANSS total

In the case of PANSS total, 17 studies reported outcomes, involving 8 different intervention measures. Initially, we assessed the consistency and inconsistency of direct and indirect comparisons among the 17 studies. The results indicated that almost all P-values were greater than 0.05, with a total P-value of 0.4205, suggesting that the consistency among the studies is acceptable.

The statistical significance results of the NMA are as follows (Table 6): MHAE [MD: − 7.90, 95% CI: (− 15.29, − 0.50)] showed a greater advantage than TUA in improving PANSS total. The SUCRA rankings for different intervention measures in improving PANSS total were as follows: MHAE (84.7%) > Yoga (79.9%) > RE (63.9%) (Fig. 4D).

**Table 6:** League table on PANSS total.

Yoga	MHAE	RE	SE	MM	CTS	TUA	LAE
Yoga	− 0.04 (− 11.19, 11.11)	2.45 (− 13.43, 18.34)	5.30 (− 11.46, 22.06)	7.39 (− 5.95, 20.73)	7.25 (− 4.18, 18.68)	7.85 (− 0.28, 15.98)	10.19 (− 2.69, 23.06)
0.04 (− 11.11, 11.19)	MHAE	2.50 (− 13.03, 18.02)	5.35 (− 7.07, 17.76)	7.43 (− 9.95, 24.81)	7.29 (− 4.67, 19.25)	7.90 (0.50, 15.29)	10.23 (− 4.58, 25.03)
− 2.45 (− 18.34, 13.43)	− 2.50 (− 18.02, 13.03)	RE	2.85 (− 17.06, 22.76)	4.94 (− 15.81, 25.68)	4.80 (− 11.76, 21.35)	5.40 (− 8.25, 19.05)	7.73 (− 11.02, 26.48)
− 5.30 (− 22.06, 11.46)	− 5.35 (− 17.76, 7.07)	− 2.85 (− 22.76, 17.06)	SE	2.09 (− 19.33, 23.51)	1.95 (− 15.34, 19.23)	2.55 (− 11.95, 17.05)	4.88 (− 14.47, 24.23)
− 7.39 (− 20.73, 5.95)	− 7.43 (− 24.81, 9.95)	− 4.94 (− 25.68, 15.81)	− 2.09 (− 23.51, 19.33)	MM	− 0.14 (− 17.70, 17.42)	0.46 (− 15.16, 16.08)	2.80 (− 15.74, 21.33)
− 7.25 (− 18.68, 4.18)	− 7.29 (− 19.25, 4.67)	− 4.80 (− 21.35, 11.76)	− 1.95 (− 19.23, 15.34)	0.14 (− 17.42, 17.70)	CTS	0.60 (− 8.77, 9.97)	2.93 (− 8.87, 14.74)
− 7.85 (− 15.98, 0.28)	− 7.90 (− 15.29, − 0.50)	− 5.40 (− 19.05, 8.25)	− 2.55 (− 17.05, 11.95)	− 0.46 (− 16.08, 15.16)	− 0.60 (− 9.97, 8.77)	TUA	2.33 (− 10.52, 15.18)
− 10.19 (− 23.06, 2.69)	− 10.23 (− 25.03, 4.58)	− 7.73 (− 26.48, 11.02)	− 4.88 (− 24.23, 14.47)	− 2.80 (− 21.33, 15.74)	− 2.93 (− 14.74, 8.87)	− 2.33 (− 15.18, 10.52)	LAE

### Publication bias test

**Figure 5:**
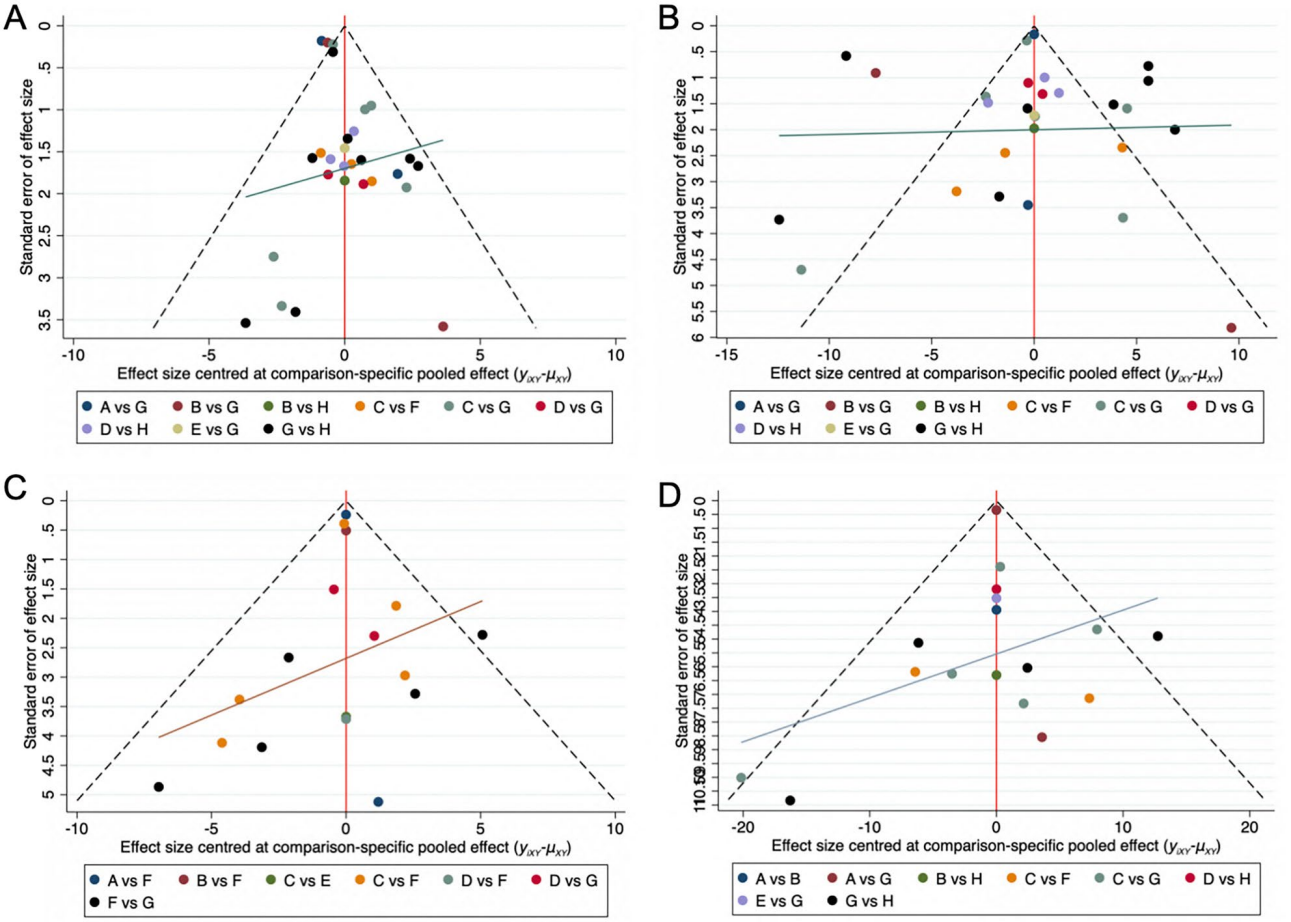
Funnel plot on publication bias. (**A**) Positive symptoms. (**B**) Negative symptoms. (**C**) General psychopathology. (**D**) PANSS total.

In order to investigate potential publication bias, we conducted Egger and Begg tests for each outcome variable and created funnel plots (Fig. 5). For all four outcome indicators, the P-values of Egger and Begg tests were greater than 0.05, and the funnel plots showed no apparent publication bias^62^.

## Discussion

To determine the optimal exercise modality for treating schizophrenia, this NMA compared the effectiveness of 8 different interventions based on 25 randomized controlled trials. The study found that RE and Yoga have a more positive impact on treating Positive Symptoms. Yoga and LAE are more effective in treating Negative Symptoms. LAE and MHAE are considered better exercise modalities for treating General Psychopathology. MHAE and Yoga demonstrate a more positive effect in treating PANSS total. The results indicate that, among the outcome indicators for schizophrenia, there is no single most effective exercise modality, and different interventions may be more effective for different outcomes.

The classification of exercise types is a key aspect of this study. A detailed categorization of exercise types is instrumental in gaining a deeper understanding of schizophrenia treatment, and provides more precise and actionable guidance for clinical practice. In the studies included, we classified exercise interventions into 8 categories. We singled out Yoga as a separate category mainly because of the large number of Yoga studies, with the majority originating from India and Japan. Additionally, Yoga’s focus is not primarily on improving cardiovascular endurance. Although it may include some aerobic movements and fluid movement sequences, it emphasizes body flexibility, balance, core strength, and meditative states. Aerobic exercise was categorized into two types: medium to high-intensity aerobic exercise and low-intensity aerobic exercise. The basis for this classification is the combination of exercise type and intensity. However, some studies did not provide detailed information on exercise interventions, making precise classification challenging. Furthermore, factors such as intervention duration, frequency, and timing were not considered in the classification, thus the impact of exercise dosage needs to be carefully considered when analyzing study results. However, addressing this issue comprehensively in the current study proved to be difficult, and future research needs to delve deeper into this viewpoint.

Furthermore, in the research on Positive Symptoms, although only one literature involved RE^28^, based on the results of that literature, RE was considered the most effective exercise modality. Previous studies have utilized RE as an intervention for treating schizophrenia^63,64^; however, unfortunately, the required outcome indicators were not included in their results, leading to their exclusion from our analysis. Research on the effectiveness of RE in treating schizophrenia is limited, and more studies are needed to confirm its effects. On the other hand, Yoga has shown significant therapeutic effects in improving Positive Symptoms, as indicated by the League table, demonstrating its superior effectiveness compared to CTS, LAE, and MM.

The research results regarding Negative Symptoms indicate that Yoga is the most effective exercise modality. This conclusion aligns with the findings of Meyer et al.^65^ and Broderick et al.^66^, collectively affirming Yoga as an effective exercise therapy for treating mental illnesses. However, Yoga interventions in studies predominantly originate from India and Japan, and the effective confirmation of regional variations remains pending. Nevertheless, based on published research, Yoga undeniably demonstrates significant improvements in Schizophrenia symptoms. Additionally, our study results highlight the positive impact of LAE on improving Negative Symptoms. Vogel et al.’s meta-analysis indicates a significant influence of aerobic exercise on the negative symptoms of schizophrenia^27^, although the study does not specify differences related to aerobic exercise intensity.

In the outcomes of General Psychopathology and PANSS total, we observe that LAE, MHAE, and yoga were the preferable types of exercise. This finding shares some similarity with previous research. Brendon Stubbs et al. explored the intensity of exercise on the basis of exercise therapy for schizophrenia and concluded that moderate to vigorous exercise intensity is more beneficial^67^. Similarly, Nicole Korman et al. suggested that moderate to vigorous Aerobics has significant improvements in certain functions for individuals with schizophrenia^68^. Our study indicates that different intensities of aerobic exercise have positive effects on schizophrenia, albeit possibly targeting different aspects of improvement.

In summary of the results from this study, we believe that Yoga, aerobic exercise, and RE are all effective exercise therapies worth promoting and applying. However, their impact may vary to some extent depending on different outcome indicators. Future research can delve deeper into the therapeutic mechanisms of these exercise modalities and provide additional scientific evidence.

## Strengths and limitations

Firstly, this NMA represents the first attempt to compare the effectiveness of different types of exercise on schizophrenia, providing reliable evidence for the optimal exercise interventions with scientific value in alleviating symptoms of schizophrenia. Secondly, we rigorously adhered to the evidence-based recommendations for NMA, ensuring the authenticity and reliability of the study results.

However, there are still some limitations to our NMA. Firstly, we excluded cross-sectional and observational studies, resulting in a limited number of available studies, which might impact the reliability of the results. Secondly, due to the constraints of NMA, we were unable to incorporate additional information such as intervention frequency, duration, and intensity, limiting the analysis to a general level. Future research should involve larger and more comprehensive clinical studies on exercise, aiming to gather sufficient evidence for a more direct and comprehensive comparison of the efficacy of exercise in treating schizophrenia.

## Conclusions

Our study indicates that among different types of exercise, LAE emerges as the optimal intervention for improving General Psychopathology, and Yoga demonstrates the best efficacy in enhancing PANSS total scores in patients with schizophrenia. Moreover, RE seems to be effective in improving Positive Symptoms, while Yoga is more effective in alleviating Negative Symptoms.

### Supplementary Information

Supplementary Information.

## Data Availability

The original contributions presented in the study are included in the article/Supplementary Material, further inquiries can be directed to the corresponding author.

## Abbreviations

CTS: Chinese traditional sports
RE: Resistance exercise
SE: Stretching exercise
LAE: Low-intensity aerobic exercise
MD: Mean differences
MHAE: Medium and high-intensity aerobic exercise
MM: Multi-mode movement
NMA: Network Meta-Analysis
PANSS: Positive And Negative Syndrome Scale for Schizophrenia
SANS: Scale for Assessment of Negative Symptoms
SAPS: Scale for Assessment of Positive Symptoms
SUCRA: Surface Under the Cumulative Ranking
SD: Standard deviations
TAU: Treatment-as-usual
WHO: World Health Organization
95% CI: 95% Confidence interval
