# Supplementary material for: Comparative efficacy of different types of exercise modalities on psychiatric symptomatology in patients with schizophrenia: a systematic review with network meta-analysis
Source: Sci Rep. 2024 Mar 25;14:7019. doi: 10.1038/s41598-024-57081-3 (PMC10963726; doi:10.1038/s41598-024-57081-3)
Supplement: Supplementary file 1 — Supplementary Information. [file 41598_2024_57081_MOESM1_ESM.pdf]

Supplementary Materials:

Appendix S1 Search Strategy

Database	Search strategy	amount
Pubmed		
#1	Search: "Exercise"[Mesh] Sort by: Most Recent	245477
#2	Search: ((((((((((((((((((((Exercises[Title/Abstract]) OR (Sports[Title/Abstract])) OR (Training[Title/Abstract])) OR (Physical Activity[Title/Abstract])) OR (Acute Exercise[Title/Abstract])) OR (Aerobic exercise[Title/Abstract])) OR (Isometric Exercises[Title/Abstract])) OR (Nerve Exercise[Title/Abstract])) OR (Leisure Activities[Title/Abstract])) OR (Endurance[Title/Abstract])) OR (Resistance[Title/Abstract])) OR (Flexibility[Title/Abstract])) OR (Sports games[Title/Abstract])) OR (Running[Title/Abstract])) OR (Bicycles[Title/Abstract])) OR (Gymnastics[Title/Abstract])) OR (Jump rope[Title/Abstract])) OR (Dance[Title/Abstract])) OR (Tai Chi[Title/Abstract])) OR (Yoga[Title/Abstract])) OR (Ball Sports[Title/Abstract])) OR (Racquet Sports[Title/Abstract])) OR (Water Sports[Title/Abstract])) OR (Swimming[Title/Abstract])	1844810
#3	#1 OR #2	1940397
#4	Search: "Schizophrenia"[Mesh] Sort by: Most Recent	114957
#5	Search: (((((Schizophrenias[Title/Abstract]) OR (Schizophrenic Disorders[Title/Abstract])) OR (Disorder, Schizophrenic[Title/Abstract])) OR (Disorders, Schizophrenic[Title/Abstract])) OR (Schizophrenic Disorder[Title/Abstract])) OR (Dementia Praecox[Title/Abstract])	2140
#6	#4 OR #5	115452

#7	#3 AND #6	4088
embase		
#1	('exercise'/exp OR exercise OR exercises:ab,ti OR sports:ab,ti OR training:ab,ti OR 'physical activity':ab,ti OR 'acute exercise':ab,ti OR 'aerobic exercise':ab,ti OR 'isometric exercises':ab,ti OR 'nerve exercise':ab,ti OR 'leisure activities':ab,ti OR endurance:ab,ti OR resistance:ab,ti OR flexibility:ab,ti OR 'sports games':ab,ti OR running:ab,ti OR bicycles:ab,ti OR gymnastics:ab,ti OR 'jump rope':ab,ti OR dance:ab,ti OR 'tai chi':ab,ti OR yoga:ab,ti OR 'ball sports':ab,ti OR 'racquet sports':ab,ti OR 'water sports':ab,ti OR swimming:ab,ti) AND ([controlled clinical trial]/lim OR [randomized controlled trial]/lim)	149969
#2	('schizophrenia'/exp OR schizophrenia OR schizophrenias:ab,ti OR 'schizophrenic disorders':ab,ti OR 'disorder, schizophrenic':ab,ti OR 'disorders, schizophrenic':ab,ti OR 'schizophrenic disorder':ab,ti OR 'dementia praecox':ab,ti) AND ([controlled clinical trial]/lim OR [randomized controlled trial]/lim)	11683
#3	#1 AND #2	1453
web of science		
#1	((((((((((((((((((((((TS=(Exercise)) OR TS=(Exercises)) OR TS=(Sports)) OR TS=(Training)) OR TS=(Physical Activity)) OR TS=(Acute Exercise)) OR TS=(Aerobic exercise)) OR TS=(Isometric Exercises)) OR TS=(Nerve Exercise)) OR TS=(Leisure Activities)) OR TS=(Endurance)) OR TS=(Resistance)) OR TS=(Flexibility)) OR TS=(Sports games)) OR TS=(Running)) OR TS=(Bicycles)) OR TS=(Gymnastics)) OR TS=(Jump rope)) OR TS=(Dance)) OR TS=(Tai Chi)) OR TS=(Yoga)) OR TS=(Ball Sports)) OR TS=(Racquet Sports)) OR TS=(Water Sports)) OR TS=(Swimming)	5144149
#2	((((((TS=(Schizophrenia)) OR TS=(Schizophrenias)) OR TS=(Schizophrenic Disorders)) OR TS=(Disorder, Schizophrenic)) OR TS=(Disorders, Schizophrenic)) OR TS=(Schizophrenic Disorder)) OR TS=(Dementia Praecox)	206406

#3	#2 AND #1	13023
#4	((((TS=(Randomized controlled trial)) OR TS=(controlled clinical trial)) OR TS=(randomized)) OR TS=(placebo)) OR TS=(randomly)	1629693
#5	#3 AND #4	2430
Cochrane		
#1	(Exercise):ti,ab,kw OR (Exercises):ti,ab,kw OR (Sports):ti,ab,kw OR (Training):ti,ab,kw OR (Physical Activity):ti,ab,kw	260513
#2	(Acute Exercise):ti,ab,kw OR (Aerobic exercise):ti,ab,kw OR (Isometric Exercises):ti,ab,kw OR (Nerve Exercise):ti,ab,kw OR (Leisure Activities):ti,ab,kw	35842
#3	(Endurance):ti,ab,kw OR (Resistance):ti,ab,kw OR (Flexibility):ti,ab,kw OR (Sports games):ti,ab,kw OR (Running):ti,ab,kw	138440
#4	(Bicycles):ti,ab,kw OR (Gymnastics):ti,ab,kw OR (Jump rope):ti,ab,kw OR (Dance):ti,ab,kw OR (Tai Chi):ti,ab,kw	9585
#5	(Yoga):ti,ab,kw OR (Ball Sports):ti,ab,kw OR (Racquet Sports):ti,ab,kw OR (Water Sports):ti,ab,kw OR (Swimming):ti,ab,kw	7608
#6	#1 OR #2 OR #3 OR #4 OR #5	357371
#7	(Schizophrenia):ti,ab,kw OR (Schizophrenias):ti,ab,kw OR (Schizophrenic Disorders):ti,ab,kw OR (Disorder, Schizophrenic):ti,ab,kw OR (Disorders, Schizophrenic):ti,ab,kw	19081
#8	(Schizophrenic Disorder):ti,ab,kw OR (Dementia Praecox):ti,ab,kw	1906
#9	#7 OR #8	19083

#10	#6 AND #9	4363
